# Supplementary material for: Detection and Spatio-Temporal Distribution of Pinnatoxins in Shellfish from the Atlantic and Cantabrian Coasts of Spain
Source: Toxins (Basel). 2019 Jun 14;11(6):340. doi: 10.3390/toxins11060340 (PMC6628396; doi:10.3390/toxins11060340)
Supplement: Supplementary file 1 [file toxins-11-00340-s001.pdf]

Supplementary material: Detection and spatio-temporal distribution of pinnatoxins in shellfish from the Atlantic and Cantabrian coasts of Spain

J. Pablo Lamas, Fabiola Arévalo, Ángeles Moroño, Jorge Correa, Susana Muñíz and Juan Blanco

Table S1. ANOVA and TukeyHSD post-hoc tests of the differences in PnTX G concentration between habitats (Wild mussel – Raft mussel) and between species, using logarithmically transformed data.

```
LMlogespecies<-lm(log(Pinna.G)~ESPECIE2,data=pinna)
> AOVlogespecies=aov(LMlogespecies)
> summary(AOVlogespecies)
```

	Df	Sum Sq	Mean Sq	F value	Pr(>F)
ESPECIE2	5	27.34	5.467	14.39	2.87e-12
Residuals	231	87.77	0.380	-	-

Signif. codes: 0 ‘***’ 0.001 ‘**’ 0.01 ‘*’ 0.05 ‘.’ 0.1 ‘ ’ 1
544 observations deleted due to missingness

```
> TukeyHSD(AOVlogespecies)
```

Tukey multiple comparisons of means
95% family-wise confidence level

Fit: aov(formula = LMlogespecies)

$ESPECIE2

	diff	lwr	upr	p adj
Wild mussel-Raft mussel	0.7867092	0.4937221	1.07969617	0.0000000
E.siliqua-Raft mussel	-0.0776424	-1.8539975	1.69871274	0.9999956

P. rhomboides-Raft mussel	1.9849530	0.2085978	3.76130811	0.0186416
Vp. corrugata-Raft mussel	0.7231904	-0.1723991	1.61877983	0.1900166
C. edule-Raft mussel	0.0387578	-0.6438011	0.72131669	0.9999838
E.siliqua-Wild mussel	-0.8643516	-2.6548885	0.92618543	0.7346804
P. rhomboides-Wild mussel	1.1982438	-0.5922932	2.98878079	0.3906575
Vp. corrugata-Wild mussel	-0.0635188	-0.9869177	0.85988015	0.9999580
C. edule-Wild mussel	-0.7479514	-1.4666110	-0.02929167	0.0360024
P. rhomboides-E.siliqua	2.0625954	-0.4425232	4.56771390	0.1726591
Vp. corrugata-E.siliqua	0.8008328	-1.1796373	2.78130285	0.8543170
C. edule-E.siliqua	0.1164002	-1.7772914	2.01009181	0.9999760
Vp. corrugata-P. rhomboides	-1.2617626	-3.2422327	0.71870748	0.4479777
C. edule-P. rhomboides	-1.9461952	-3.8398868	-0.05250356	0.0400554
C. edule-Vp. corrugata	-0.6844326	-1.7947077	0.42584256	0.4861949

Table S2. Regression analysis of the relationship between % of esterification and total PnTX G in mussels, with logarithmically transformed data.

> REGPorcEst_TotalMejLog<-lm(log10(PercEsterif)~log10(Pinna.Hidro), data= pinnaHidrolMej)
> summary(REGPorcEst_TotalMejLog) # casi significativo pero no

Call:
lm(formula = log10(PercEsterif) ~ log10(Pinna.Hidro), data = pinnaHidrolMej)

Residuals:

Min	1Q	Median	3Q	Max
-0.31066	-0.05422	0.04196	0.10338	0.31915

Coefficients:

	Estimate Std.	Error t	value	Pr(>|t|)
(Intercept)	0.9333	0.1639	5.693	7.38e-05
log10(Pinna.Hidro)	0.3655	0.2031	1.799	0.0953

Signif. codes: 0 '***' 0.001 '**' 0.01 '*' 0.05 '.' 0.1 ' ' 1

Residual standard error: 0.1743 on 13 degrees of freedom
Multiple R-squared: 0.1993, Adjusted R-squared: 0.1377
F-statistic: 3.236 on 1 and 13 DF, p-value: 0.09527
